# Multiple orthokeratinized odontogenic cysts: clinical, pathological, and genetic characteristics

**DOI:** 10.1186/s13000-022-01261-0

**Published:** 2022-10-14

**Authors:** Sawako Ono, Katsutoshi Hirose, Shintaro Sukegawa, Satoko Nakamura, Daisuke Motooka, Yuri Iwamoto, Yumiko Hori, Kaori Oya, Yasuo Fukuda, Satoru Toyosawa

**Affiliations:** 1 Department of Pathology, Kagawa Prefectural Central Hospital, 1-2-1 Asahimachi, 760-8557 Takamatsu, Kagawa Japan; 2 Department of Pathology and Medicine, Graduate School of Medicine, Dentistry and Pharmaceutical Sciences, Okayama University, Okayama, Japan; 3 Department of Oral Pathology, Osaka University Graduate School of Dentistry, 1-8 Yamadaoka, 565-0871 Suita, Osaka Japan; 4 Department of Oral and Maxillofacial Surgery, Kagawa Prefectural Central Hospital, 1-2-1 Asahimachi, 760-8557 Takamatsu, Kagawa Japan; 5 Department of Oral and Maxillofacial Surgery, Kagawa University School of Medicine, 1750-1 Ikenobe, 761-0793 Miki, Kagawa Japan; 6 Genome Information Research Center, Research Institute for Microbial Diseases, Osaka University, 3-1 Yamadaoka, 565-0871 Suita, Osaka Japan; 7 Department of Oral and Maxillofacial Radiology, Osaka University Graduate School of Dentistry, 1-8 Yamadaoka, 565-0871 Suita, Osaka Japan; 8 Department of Pathology, Osaka University Graduate School of Medicine, 2-2 Yamadaoka, 565-0871 Suita, Osaka Japan; 9 Department of Central Laboratory and Surgical Pathology, National Hospital Organization, Osaka National Hospital, 2-1-14 Hoenzaka, 540-0006 Osaka, Japan; 10 Division for Clinical Laboratory, Osaka University Dental Hospital, 1-8 Yamadaoka, 565-0871 Suita, Osaka Japan

**Keywords:** Orthokeratinized odontogenic cyst, Odontogenic keratocyst, Odontogenic cyst, Odontogenic tumor, Protein patched homolog 1

## Abstract

**Background:**

Orthokeratinized odontogenic cyst (OOC) is a rare developmental odontogenic cyst of the jaw. It was originally believed to be a variant of odontogenic keratocyst (OKC) but is now considered to be a distinct entity. OOC usually presents as a single lesion and recurs infrequently. On the other hand, OKC often presents with multiple lesions and displays locally aggressive behavior and a high recurrence rate associated with the *protein patched homolog 1* (*PTCH1*) gene mutation. Multiple OOC cases are extremely rare and seem to be aggressive, but their pathogenesis is not fully understood. This study aimed to determine the clinical, pathological, and genetic characteristics of multiple OCC.

**Methods:**

Three cases of multiple OOC were evaluated for clinical and histological findings, and immunohistochemical expression of Ki-67 and Bcl-2. Furthermore, *PTCH1* mutations were analyzed by next-generation sequencing using a custom panel to cover the entire exon of *PTCH1*.

**Results:**

The three cases of multiple OOC included two men and one woman with a mean age of 25.3 years old (range, 18–38 years old). Each case had two or three OOCs (total of seven OOCs), all of which were simultaneously detected. Of the seven OOCs that manifested as multiple jaw cysts, seven (100%) occurred in the posterior regions, four (57.1%) occurred in the mandible, and four (57.1%) were associated with an impacted tooth. Histological examination revealed cysts lined by orthokeratinized stratified squamous epithelium. Immunohistochemistry showed a low Ki-67 labeling index and no Bcl-2 expression in the seven OOCs. No pathogenic *PTCH1* mutations were detected in any of the seven OOCs. None of the patients had any other symptoms or signs of recurrence at the last follow-up (6–60 months).

**Conclusion:**

Multiple OOCs appeared to occur more often in younger patients than solitary OOC. Both multiple and solitary OOCs may be related diseases within the entity of odontogenic cysts. Multiple OOCs are clinicopathologically and genetically distinct from OKC.

## Introduction

Orthokeratinized odontogenic cyst (OOC) is a developmental odontogenic cyst characterized by a lining of orthokeratinized stratified squamous epithelium [1]. In 1981, they were first described by Wright et al. [2], and were originally thought to be in the spectrum of odontogenic keratocysts (OKC). However, several studies have discussed the clinical and pathological differences between OOC and OKC [2–9]. The new edition of the World Health Organization classification of head and neck tumors published in 2022 has described OOC as a distinct entity from OKC [1, 10].

Histologically, OOC shows predominantly orthokeratinization with a granular cell layer, whereas OKC displays parakeratinization with corrugated surface keratin layers and palisaded basal cells [1]. Clinically, OOC recurs less frequently, in comparison to OKC, which has recurrence rate as high as 2.5–62% [1, 9, 11]. In addition, OKC is genetically associated with mutations in the *protein patched homolog 1* (*PTCH1*) gene, which activates the Sonic hedgehog (SHH) signaling pathway and results in aberrant cell proliferation of the OKC epithelium [1, 10, 11]. The *PTCH1* mutation was detected in up to 93% of sporadic OKCs [10]. In addition, 5% of all OKCs occur as part of nevoid basal cell carcinoma syndrome (NBCCS) caused by *PTCH1* mutation [1, 11]. However, pathogenic *PTCH1* mutations have not been detected in OOC [9, 12].

OOC usually presents as a single lesion, whereas about 10% of OKC present with multiple lesions [1, 9, 11]. Multiple OOC are extremely rare, and only 18 cases of multiple OOC have been reported in the literature to date [9, 13–19]. However, little is known about the differences in clinicopathological and genetic characteristics of multiple OOC between solitary OOC and OKC. Here, we report the results of the clinical, histological, immunohistochemical, and genetic analyses of a small series of multiple OOC cases.

## Methods

### Histological and immunohistochemical examination

Three cases of multiple OOC with formalin-fixed paraffin-embedded (FFPE) tissues were retrieved from the pathology files. The final diagnosis of OOC was made by two pathologists (SO and KH). This study was approved by the ethical review board of the Graduate School of Dentistry, Osaka University (IBR No. R1-E46 and R4-E1).

Resected tissue samples were fixed with 10% formalin, routinely embedded in paraffin, cut into 4 μm thick serial sections, and used for hematoxylin and eosin, and immunohistochemical staining. Immunohistochemical staining was performed using a Roche Ventana BenchMark GX autostainer (Ventana Medical Systems, Tucson, AZ, USA) according to the manufacturer’s instructions. Primary antibodies against Ki-67 and Bcl-2 were used.

### Next-generation sequencing (NGS)

To examine *PTCH1* mutational status, we performed next-generation sequencing (NGS) with a custom panel as previously described [20]. Genomic DNA was extracted from FFPE tissues using the QIAamp DNA FFPE Tissue Kit (Qiagen, Valencia, CA, USA) according to the manufacturer’s instructions. The gene panel was designed using SureDesign [21] to cover the whole exon of *PTCH1* gene (NM_000264.3). On average, 70 ng of the extracted DNA was fragmented using the SureSelect Fragmentation Enzyme (Agilent Technologies, Inc. Santa Clara, CA, USA) to 150–200 bp. Sequence libraries were prepared using a custom SureSelect Low Input Target Enrichment System (Agilent Technologies Inc. Santa Clara, CA, USA), according to the manufacturer’s instructions, and sequenced using Illumina MiSeq (Illumina, San Diego, CA, USA). SureCall ver4.0 [22] was used for variant calling. DNA in introns or non-cording DNA was excluded.

## Results

### Clinical findings

**Fig. 1:**
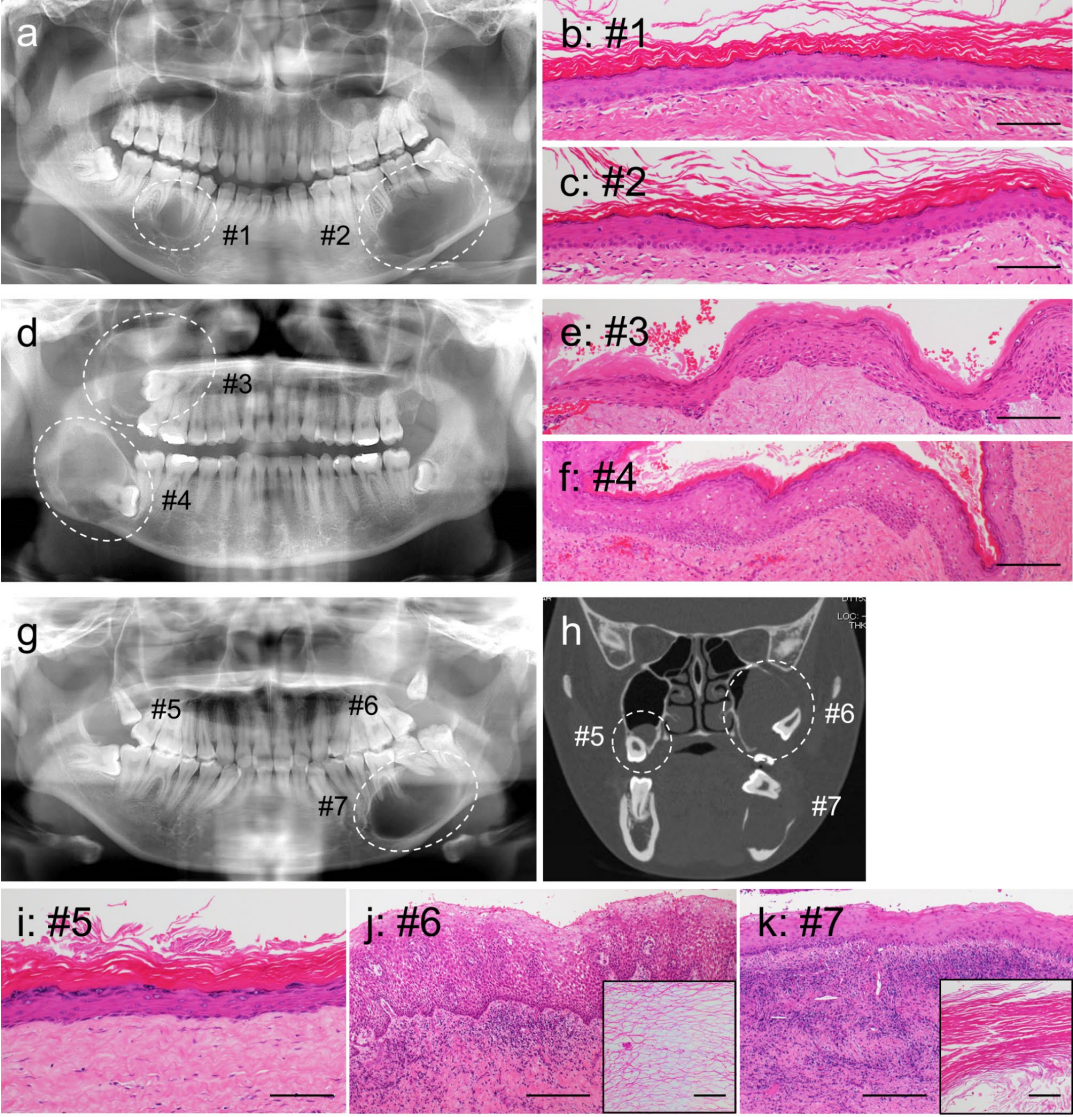
Radiographic and histological analyses of multiple OOC. Panoramic radiographs of Case 1 (**a**), 2 (**d**), and 3 (**g**), and coronal CT of Case 3 (**h**). The OOCs show a well-demarcated, unilocular lesion (dotted line). Representative histological findings in Cases 1 (**b, c**), 2 (**e, f**), and 3 (**i-k**). The epithelial lining of OOC shows orthokeratinization with prominent granular cells. The lumen was filled with keratin material (insets **i** and **k**). OOC, orthokeratinized odontogenic cysts; CT, computed tomography. Scale bars: 100 μm in Figs. b, c, e, f, i, j and k

The three cases included two men and one woman, with a mean age of 25.3 years old (range, 18–38 years old). The patients had no relevant medical or family history. The presenting symptoms were swelling without pain (Cases 1 and 2) and with pain (Case 3). The preoperative clinical diagnoses were OKC (Cases 1 and 3) and odontogenic cysts (Case 2). Each case had two or three OOCs (total of seven OOCs), all of which were detected simultaneously on radiographic examination (Fig. 1a, d, g, h). Of the seven OOCs that manifested as multiple jaw cysts, seven (100%) occurred in the posterior regions, four (57.1%) occurred in the mandible, and four (57.1%) were associated with an impacted tooth. All patients were treated with enucleation and had no signs of recurrence at the last follow-up (6–60 months). At the last follow-up, no clinical features of NBCCS were noted. The clinical findings of multiple OOC in previously reported cases and in the present series are summarized in Table 1.

**Table 1:** Clinical findings of multiple orthokeratinized odontogenic cysts in previous reported cases and present series

Study	Age (years old)	Sex	Location	Number of total cysts	Number of cysts associated with an impacted tooth	Recurrence
Premalatha et al. [13]	35	M	Bi Post Mn	2	0	NA
Pereira et al. [14]	23	F	Bi Post Mn	2	2	No (27 months)
Pimpalkar et al. [15]	23	M	Bi Post Mn	2	2	NA
Cheng et al. [16]	23	M	Bi Post Mx, Bi Post Mn	4	4	No (14 months)
Crane et al. [17]	23	M	Uni Post Mx, Bi Post Mn	3	2	No (48 months)
	20	M	Bi Post Mx	2	2	No (24 months)
Joseph et al. [18]	29	F	Uni Post Mx, Uni Post Mn	2	2	No (11 months)
Oh et al. [19]	19	M	Uni Post Mx, Uni Post Mn	2	2	No (14 months)
	20	M	Bi Post Mn	2	0	No (60 months)
	26	M	Bi Post Mn	2	2	No (30 months)
Ono et al. (present study)	20	M	Bi Post Mn	2	0	No (6 months)
	38	M	Uni Post Mx, Uni Post Mn	2	2	No (60 months)
	18	F	Bi Post Mx, Uni Post Mn	3	2	No (36 months)
Total	Mean, 24.4	M:F = 10:3	Post, 100%; Mn, 66.7%	30	22 (73.3%)	

Excluding 8 cases reported by Wang et al. [9]. Bi, bilateral; Mn, mandible; Mx, maxilla; Post, posterior; Uni, unilateral; NA, data not available

### Histological findings

All seven OOCs showed a fibrous cyst wall lined by stratified squamous epithelium of variable thickness (Cyst #1–7) (Fig. 1b, c, e, f, i-k). An orthokeratinized epithelial lining with a prominent granular cell layer was observed (Cyst #1–5) (Fig. 1b, c, e, f, i). In two cysts (Cyst #6, 7) (Fig. 1i, k), broad non-keratinization was detected in association with inflammation, but the lumen of the cyst was filled with keratin material (insets of Fig. 1i and k). Histological findings of OKC, including corrugated surface keratin layers, palisaded basal cells, or small satellite cysts in the wall, were not observed. In addition, there were no findings suggestive of other odontogenic lesions.

### Immunohistochemical findings

**Fig. 2:**
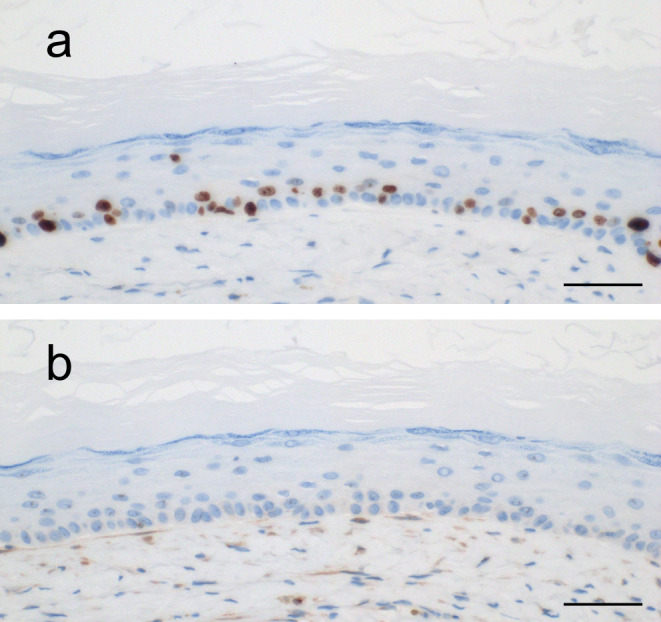
Immunohistochemical analysis of multiple OOC. Representative immunohistochemical staining patterns of Ki-67 (**a**) and Bcl-2 (**b**) in multiple OOC. OOC, orthokeratinized odontogenic cysts. Scale bars: 50 μm in Figs. a and b

Immunohistochemical examination of Ki-67 and Bcl-2 expression was performed. The Ki-67 labeling index, indicating the proliferative activity, was 9.43% (range, 6–13%) (Fig. 2a). Bcl-2 expression was not detected in any of the seven OOCs (Fig. 2b). The results of the immunohistochemical analysis are summarized in Table 2.

**Table 2:** Summary of immunohistochemical and molecular genetic findings

Case	Cyst #	Site	Ki-67 (%)	Bcl-2	*PTCH1* mutation
1	#1	Right mandible	11	-	None
	#2	Left mandible	10	-	p.P1315L
2	#3	Right maxilla	6	-	p.P1315L
	#4	Right mandible	9	-	p.P1315L
3	#5	Right maxilla	6	-	p.G1212S, p.P1315L
	#6	Left maxilla	11	-	p.G1212S
	#7	Left mandible	13	-	p.G1212S, p.P1315L

Staining intensity (-, no expression; +, positive). *PTCH1; the protein patched homolog 1*

### Molecular genetic findings

*PTCH1* mutations were evaluated in all seven OOCs. *PTCH1* mutations were detected in six lesions collected in the three analyzed cases. Of the seven OOCs, *PTCH1* mutations (p.P1315L) were identified in five OOCs (Cyst #2–5, 7), and *PTCH1* mutations (p.G1212S) were identified in three OOCs (Cyst #5–7). None of the *PTCH1* mutations were detected in one OOC (Cyst #1). The results of the molecular genetic analyses are summarized in Table 2.

## Discussion

Multiple OOCs are an extremely rare developmental odontogenic cyst of the jaw. The present study is the first to report a series of clinical, histological, immunohistochemical, and genetic analyses of synchronous multiple OOC cases. To date, a total of 21 cases with multiple OOC have been reported, including the present study (Table 1) [9, 13–19]. Wang et al. [9] reported eight cases of multiple OOC, but did not provide detailed clinical data, including that regarding age, sex, cyst location, number of cysts, and medical history. After removing these eight cases, a total of 13 cases were analyzed (Table 1). The mean age was 24.4 years old (range, 18–38 years old), and the male-to-female ratio was 10:3. The mandible (66.7%, 20/30) was 2 times more frequently affected than maxilla (33.3%; 10/30). All OOCs were located in the posterior regions of the jaw (100%, 30/30), and 22 OOCs of which were associated with impacted teeth (73.3%). No association with NBCCS was reported in any patient and no evidence of recurrence was noted (Table 1). Compared with solitary OOC and OKC in previous studies [2, 12, 19, 23, 24], the mean age of patients with multiple OCC was about 10 years younger (Multiple OOC, 24.4 years old vs. Solitary OOC, 31.5–38.9 years old vs. OKC, 32.8–33.1 years old). Both multiple and solitary OOC were predominant in men compared with OKC (Male-to-female ratio; Multiple OOC, 3.3:1 vs. Solitary OOC, 2.6:1 vs. OKC, 1.05–1.76) [19].

Histologically, OOC is characterized by an orthokeratinized stratified squamous epithelium lining with prominent granular cell [1, 2, 19]. OOC often shows varying degrees of inflammation in the cyst wall and non-keratinization is detected in association with inflammation [19]. Multiple OOC in previously reported cases and the present series showed typical histological features of OOC, but no features of OKC have been reported [13–19]. Previous studies have shown that the immunohistochemical expression of various markers related to biological behavior varies between solitary OOC and OKC [3–8]. The present series was in general agreement with previous immunohistochemical findings, and a lower proliferative activity in OOC linings was confirmed (Ki-67: Multiple OOC, 9.43% vs. Solitary OOC, 6-8.6% vs. OKC 10.7–53%) (Fig. 2a) [5, 7, 8, 11]. Unlike OKC, Bcl-2 expression was not detected in multiple OOC or in previously reported solitary OOC (Fig. 2b) [6, 9]. Bcl-2, regulated by the SHH downstream protein Gli1, encodes a protein that prevents apoptosis. Lower expression of Bcl-2 in OOC may result in mild biological behavior and a lower tendency to recur [6, 9, 11]. Therefore, a low Ki-67 labeling index and lack of Bcl-2 expression in multiple OOC indicated mild biological behavior of odontogenic cysts. As a result, multiple OOC never recur and solitary OOC rarely recurs, whereas OKC displays locally aggressive behavior and a high recurrence rate [1, 9, 11]. Considering the clinical and pathological findings, multiple OOC and solitary OOC are probably the same entity as odontogenic cysts, instead of OKC.

The *PTCH1* mutation was detected in more than 80% of OKCs, both syndromic and sporadic [1, 9–11, 25]. We detected *PTCH1* p.P1315L (c.3944 C > T) mutation in all 3 cases, and p.G1212S (c.3634G > A) mutation in 1 case. The *PTCH1* p.P1315L mutation has also been identified in solitary OOC and reported to be unrelated to the risk of basal cell carcinoma, which is one of the symptoms of NBCCS [9, 26]. Several studies have indicated that *PTCH1* p.P1315L is a nonpathogenic mutation [9, 27]. Moreover, the interpretation of *PTCH1* p.P1315L is “Benign” in ClinVar [28]. The identified *PTCH1* p.G1212S mutation is reported in the COSMIC (Catalogue of Somatic Mutations in Cancer) database, however the function of this mutation is not investigated. The interpretation of *PTCH1* p.G1212S is “Likely Benign” in ClinVar [28]. Taken together, our targeted NGS did not identify any pathogenic *PTCH1* mutations common to multiple OOC cases. Two previous multiple OOC cases failed to detect pathogenic *PTCH1* mutations by genetic testing using DNA from one blood sample or one OOC lesion [9, 17]. Thus, abnormalities related to pathogenic *PTCH1* mutations frequently detected in OKC were not observed in multiple OOC cases. The absence of pathogenic *PTCH1* mutations in multiple OOC, which was in sharp contrast to the results for OKC, suggested a different entity of odontogenic cysts.

## Conclusion

In conclusion, the present study reports a small series of multiple OOC cases and confirms their consistent clinicopathological and genetic characteristics. Multiple OOCs occurred more often in younger patients than solitary OOCs. Both multiple and solitary OOCs may be related diseases within the entity of odontogenic cysts. Multiple OOC are clinicopathologically and genetically distinct from OKC.

## Data Availability

The surgical materials and datasets analyzed in the current study are available from the corresponding author upon reasonable request.

## List of abbreviations

FFPE: formalin-fixed paraffin-embedded.
NBCCS: nevoid basal cell carcinoma syndrome.
NGS: next-generation sequencing.
OOC: orthokeratinized odontogenic cyst.
OKC: odontogenic keratocyst.
PTCH1: protein patched homolog 1.
SHH: Sonic hedgehog.
